# Usability and User Satisfaction With a Prototype Weighted Glove in People With Parkinson’s Disease: An Exploratory Secondary Analysis

**DOI:** 10.7759/cureus.117847

**Published:** 2026-10-13

**Authors:** Hriday Shah, Chaitali Shah

**Affiliations:** 1 Physiotherapy, Parul University, Vadodara, IND

**Keywords:** assistive technology, parkinson’s disease, upper-limb rehabilitation, usability, user satisfaction, wearable device, weighted glove

## Abstract

Background

Upper-limb impairments in Parkinson’s disease (PD) can affect reaching, grasping, object manipulation, and other activities required for functional independence. Wearable and glove-based rehabilitation devices may provide opportunities for upper-limb training; however, their clinical potential should be considered alongside the user’s experience. This study evaluated user-reported usability and satisfaction with a prototype weighted glove following two weeks of structured upper-limb rehabilitation in people with PD.

Methods

This exploratory secondary analysis used previously unpublished post-intervention usability data from a previously conducted single-group pretest-posttest quasi-experimental study. Fifteen people with PD who completed a two-week structured upper-limb rehabilitation programme while using the prototype weighted glove were included. Glove loading ranged from 600 to 700 g and was selected according to individual comfort, physical capacity, movement quality, and verbal feedback. The glove was worn throughout supervised rehabilitation sessions conducted six days per week for 45-60 minutes per session. Following the intervention, perceptions were assessed using the Assistive Technology Usability Questionnaire for People With Neurological Diseases (NATU Quest). Quantitative responses were summarized descriptively, and open-ended responses were reviewed descriptively for recurring areas of feedback.

Results

The mean age was 67.07 ± 7.70 years; 10 participants (66.7%) were male, and five (33.3%) were female. The mean overall NATU Quest score was 4.28 ± 0.32 out of 5. The highest-rated items were ease of learning (4.73 ± 0.46), perceived safety and security (4.67 ± 0.49), and overall satisfaction (4.60 ± 0.51). Physical comfort/fatigue received the lowest score (3.33 ± 0.72). Open-ended feedback suggested potential improvements in weight adjustability, fit, padding, fastening, fatigue, and suitability for everyday activities. All 15 participants completed the intervention and NATU Quest, and no adverse events were reported.

Conclusions

In this small exploratory cohort, the prototype weighted glove received generally high user-reported usability and satisfaction ratings after two weeks of supervised use. Comfort and fatigue were the main areas identified for further refinement. NATU Quest has no validated usability cut-off, and this study had no comparator or assessment of independent long-term use. Therefore, the findings describe short-term participant perceptions and should not be interpreted as evidence of clinical efficacy, superiority, or safety during independent use.

## Introduction

Parkinson's disease (PD) is a progressive neurodegenerative disorder characterized by bradykinesia, rigidity, tremor, and postural instability. Upper-limb impairment is particularly relevant to daily life because reaching, grasping, and manipulating objects are required for self-care and many other tasks, and difficulties with hand dexterity can reduce independence and participation [1].

Upper-limb rehabilitation is an important part of the multidisciplinary management of PD. Exercise and task-specific training have been investigated for improving hand dexterity and upper-limb function, with current evidence suggesting a small but positive effect on dexterity, although the type, intensity, and duration of interventions vary considerably [2]. This has encouraged interest in approaches that support repetitive, task-oriented practice within routine therapy. Wearable and glove-based technologies are one such approach, and previous studies have explored different mechanisms for modifying upper-limb tremor and movement in people with PD [3,4].

External loading has been investigated as a relatively simple biomechanical strategy for modifying tremor; however, evidence for tremor reduction remains limited and inconsistent. A randomized controlled trial of weighted utensils and a weighted wrist cuff found no significant reduction in postural tremor amplitude or frequency in people with PD [4], and the loads of about 600-840 g reported in earlier work were studied mainly in other tremor types [5]. Glove-based approaches using other mechanisms, such as electrical muscle stimulation, have shown that wearable glove systems for tremor management are feasible [3]. Together, these studies suggest that the design and practical characteristics of a wearable device matter alongside its potential clinical effects.

The effectiveness of a rehabilitation device does not fully describe the experience of the person using it. Ease of use, comfort, perceived safety, physical characteristics, and satisfaction may influence whether a device is acceptable and whether people are willing to keep using it. Research in people with PD has emphasized that wearable technologies should be clinically useful while also being comfortable, user-friendly, and responsive to individual needs [6], and qualitative evidence indicates that discomfort and usability difficulties may influence adoption of wearable technologies in PD [7]. User-reported assessment can therefore complement clinical outcome measures. The Assistive Technology Usability Questionnaire for People With Neurological Diseases (NATU Quest) is a 10-item questionnaire developed to assess user perceptions of assistive technology among people with neurological conditions, covering aspects such as ease of use, safety, comfort, learning, integration into daily life, and satisfaction [8].

It is not known, to our knowledge, how people with PD experience a weighted glove of about 600-700 g when it is worn throughout a multimodal upper-limb rehabilitation programme. The prototype studied here was used in a two-week programme whose clinical outcomes have been reported separately [9]. Those results do not describe how participants perceived the glove itself or which aspects of its design may need refinement. This study addresses that gap.

This study aimed to explore participants' perceptions of the usability, comfort, perceived safety, and satisfaction associated with a prototype weighted glove after two weeks of supervised upper-limb rehabilitation in people with PD and to identify aspects of the device that may require further refinement.

## Materials and methods

Study design

This study was an exploratory secondary analysis of previously unreported participant-reported data collected as a prespecified secondary outcome in a previously conducted single-group pretest-posttest quasi-experimental study. The parent study evaluated changes in tremor-related activities of daily living following a two-week structured upper-limb rehabilitation programme performed while wearing a prototype weighted glove [9].

The present analysis focused specifically on participants’ perceptions of the weighted glove, including usability, comfort, perceived safety, physical characteristics, ease of use, and overall satisfaction. These user-reported outcomes were collected using NATU Quest.

The parent study was approved by the K.J. Somaiya College of Physiotherapy Institutional Ethics Committee (Reference No. KJSCPT/264/24-25; approved July 25, 2024) and was registered with the Clinical Trials Registry of India (CTRI/2024/10/075200) [9].

The study was conducted in accordance with the principles of the Declaration of Helsinki, and written informed consent was obtained from all participants. Participants were informed of their right to withdraw from the study at any time, and the confidentiality of participant information was maintained throughout the study [9,10].

The present manuscript reports a distinct analysis of previously unpublished NATU Quest data from the same participant cohort. The clinical and functional outcomes reported in the parent publication were not re-analysed in the present study.

Study setting

The study was conducted at a private physiotherapy and rehabilitation centre in Mira Road, Mumbai, Maharashtra, India. Participants were enrolled from the local community and were recruited through the study site. Individuals diagnosed with PD who met the predefined eligibility criteria were screened for participation. The intervention sessions and all study-related assessments were conducted at the same physiotherapy and rehabilitation centre in Mumbai. Data collection for the parent study was carried out from October 21, 2024, to April 30, 2025 [9].

For the present analysis, NATU Quest data collected following completion of the two-week intervention were used (see Appendix). These data had not been reported in the previous publication.

Participants

Participants were recruited into the parent study by purposive sampling from patients with PD attending the physiotherapy rehabilitation centre. The present analysis involved no further participant selection. All 15 participants who completed the intervention also completed the post-intervention NATU Quest, and all were included in the present analysis; no participant was excluded or selected on the basis of their questionnaire responses.

Participants were eligible for inclusion in the parent study if they had a clinical diagnosis of PD, were classified within Hoehn and Yahr stages 1-3, were able to understand and follow the study procedures, and had adequate cognitive and mental status as determined by a neurologist [11].

Individuals were excluded if they had significant cognitive impairment, severe upper-limb or other joint pathology that could interfere with participation, an acute upper-limb musculoskeletal injury, another neurological condition that could compromise participation, or a systemic medical condition that could interfere with safe participation [9].

All 15 participants enrolled in the parent study completed the intervention and the post-intervention NATU Quest; no usability data were missing.

Prototype weighted glove

The prototype weighted glove was developed as a wearable upper-limb rehabilitation device intended to provide external loading during therapeutic and functional activities. The design incorporated considerations of hand mobility, weight distribution, fit, comfort, and ease of use.

The glove was constructed primarily from neoprene, selected for its flexibility and ability to conform to different hand dimensions. Spandex was incorporated around the distal openings to provide a close fit around the exposed finger regions. Multiple small, polygonal stainless-steel components with rounded or curved edges were incorporated into the glove to provide external loading rather than using a single rigid weight. The use of multiple smaller components was intended to permit greater flexibility during hand opening and closing and to distribute the load across the glove.

The metallic components were covered with foam padding and an additional neoprene layer to reduce direct contact between the metallic components and the participant’s hand. Additional stainless-steel components were positioned around the wrist region. Ball bearings were incorporated along the ulnar aspect of the palm as part of the device design to facilitate sliding contact during selected hand activities.

The first and second fingers were intentionally left uncovered to allow relatively unrestricted movement of the index and middle fingers, whereas the ring and little fingers were covered to provide additional stability. The loading capacity of the glove could be modified by varying the number and/or configuration of the stainless-steel components. The design was capable of providing approximately 450-700 g of external loading.

At the start of the programme, the supervising physiotherapist selected a glove weight of 600, 650, or 700 g for each participant according to the participant’s comfort, physical capacity, quality of movement with the glove, and verbal feedback. The selected weight was maintained throughout the two-week programme. Three participants used 600 g, six used 650 g, and six used 700 g.

The 600-700 g range was selected in accordance with previous work investigating external loading for tremor. Earlier work on wrist weighting reported a load of approximately 600-840 g in relation to tremor and fatigue, while a study of wrist weighing using Velcro weight cuffs investigated external loading in people with cerebellar tremor [5]. However, evidence regarding external loading in PD is mixed. A randomized controlled trial of weighted utensils and a weighted wrist cuff found no significant reduction in postural tremor amplitude or frequency in people with PD [4]. Therefore, the appropriateness of this loading range for people with PD remains uncertain and requires further investigation. A photograph of the prototype weighted glove with labelled components is shown in Figure 1.

**Figure 1:**
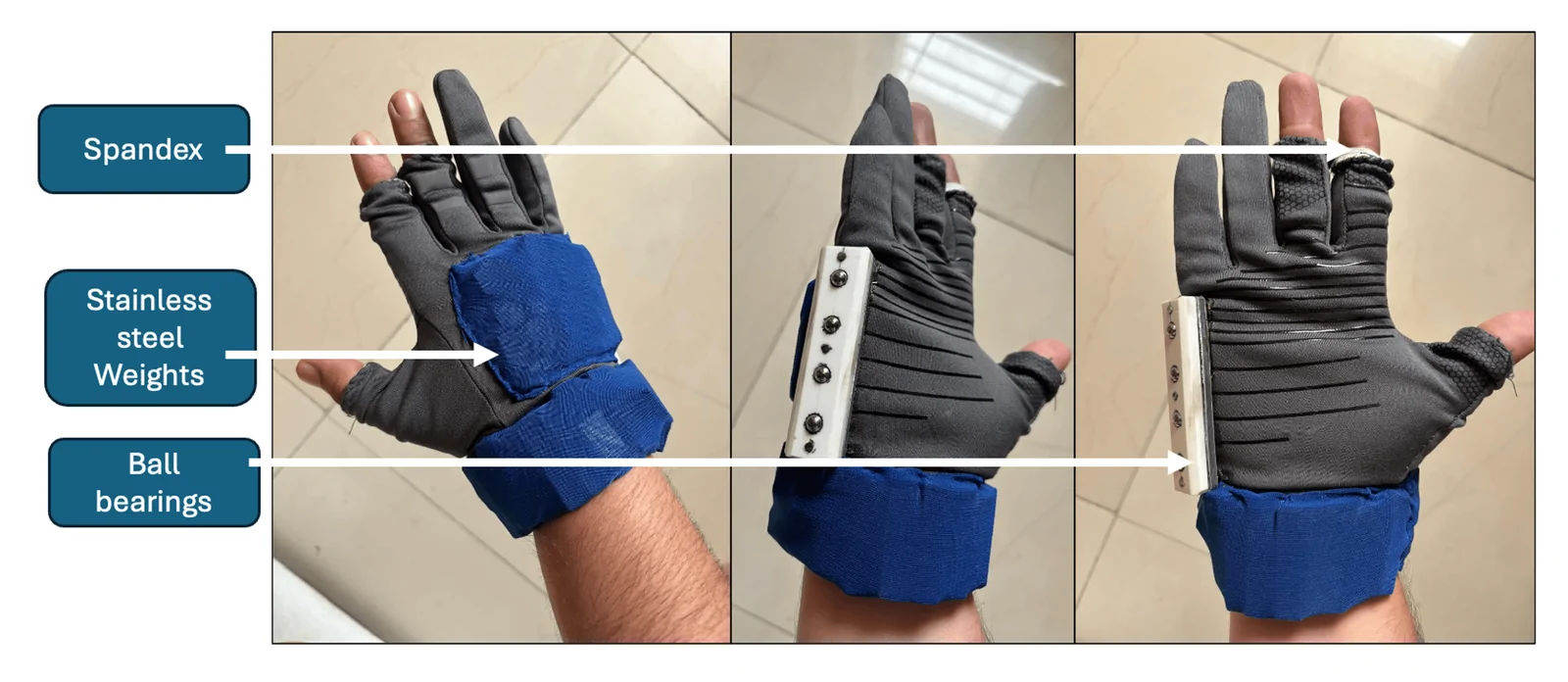
Photograph of Prototype Weighted Glove

Intervention protocol

Participants wore the weighted glove while completing a structured upper-limb rehabilitation programme for two weeks. Sessions were conducted six days per week, with each session lasting approximately 45-60 minutes. The intervention was supervised by a qualified physiotherapist and followed the rehabilitation protocol used in the parent study [9].

Each session consisted of six components: warm-up exercises, flexibility exercises, grip-strengthening exercises, resistance training, non-contact boxing, and task-oriented functional training. The weighted glove was worn during the prescribed therapeutic and functional activities throughout the intervention period [9].

The prototype weighted glove was worn throughout the entire rehabilitation session, including all prescribed therapeutic and functional activities.

Assessment of usability and user satisfaction

Following completion of the two-week rehabilitation programme, participants’ perceptions of the weighted glove were assessed using the Assistive Technology Usability Questionnaire for People With Neurological Diseases (NATU Quest) [8].

NATU Quest is a 10-item questionnaire developed to assess the perceived usability of assistive technologies among individuals with neurological conditions.

NATU Quest was developed in Spain through a two-round Delphi study involving neurorehabilitation professionals and people with neurological diseases, followed by content validation using the content validity ratio [8]. In a validation sample of 51 people with neurological diseases using assistive products, internal consistency was good (Cronbach’s alpha = 0.895), and test-retest reliability over 15 days was good (ICC = 0.869, 95% CI 0.781-0.923; Spearman’s rho = 0.818). Weighted kappa for individual items ranged from moderate to considerable agreement, and total scores correlated strongly with QUEST 2.0 (rho = 0.756). All 10 items fitted the Rasch model, with infit MNSQ values of 0.64-1.43 and outfit MNSQ values of 0.52-1.49 [8].

The 10 items assess different aspects of the user experience, including ease of use, whether the device meets the user’s needs, perceived safety, appropriateness of size, weight, and dimensions, ease of interaction with or control of the device, physical comfort/fatigue, ease of learning, willingness to recommend the device, integration into daily life, and overall satisfaction [8].

Each item is scored from 0 to 5, with higher scores indicating a more favourable perception. The comfort and fatigue item is worded so that a higher score indicates greater comfort and less fatigue. No items require reverse scoring.

NATU Quest does not have a validated cut-off for classifying usability as satisfactory. Therefore, scores in the present study are reported as observed participant ratings and are not interpreted against a predefined threshold.

Administration of NATU Quest

NATU Quest was administered once at the end of the two-week intervention. Twelve participants completed NATU Quest independently, whereas three participants required the items to be read aloud by the assessor because of motor limitations. Responses were recorded without prompting or influencing participants’ responses.

No specific procedures were used to reduce social-desirability bias. Ratings were obtained at the end of a therapist-supervised rehabilitation programme, which may have led participants to rate the glove more favourably.

Open-ended participant feedback

In addition to the scored items, NATU Quest includes three open-ended questions addressing participants’ views regarding design or manufacturing priorities, potential modifications, and desirable future features [8].

Open-ended responses were summarized descriptively as participant suggestions; no formal qualitative coding, theme development, or saturation assessment was undertaken.

Responses were reviewed for recurring areas of feedback related to comfort, weight, dimensions, ease of wearing or using the glove, perceived usefulness, safety, difficulties encountered, and suggested design modifications.

No verbatim quotations are presented because the feedback was summarized descriptively.

Safety and tolerance

Adverse events were defined as clinically relevant events such as pain, skin irritation, increased tremor, excessive fatigue, loss of balance, or exercise intolerance. These events were monitored by the supervising physiotherapist through observation and by asking participants about symptoms at each session/end of each session. No adverse events were reported during the parent study [9].

NATU Quest comfort and fatigue ratings were collected separately from adverse-event monitoring. Therefore, the physical comfort and fatigue score should not be interpreted as an adverse-event measure.

Statistical analysis

Statistical analysis was performed using JASP version 0.19.3 (JASP Team, University of Amsterdam, Amsterdam, the Netherlands) [9,12]. Demographic and clinical characteristics were summarized using descriptive statistics. Continuous variables were expressed as mean ± standard deviation or median with interquartile range, as appropriate, while categorical variables were presented as frequencies and percentages.

For NATU Quest, the overall score and individual item scores were calculated according to the instrument’s scoring procedure [7]. All 15 participants answered all 10 items; therefore, there were no missing item responses. The overall score was calculated as the mean of the 10 item scores for each participant. Individual item scores and the overall score are reported as mean ± standard deviation.

Given the exploratory nature of the present analysis and the small sample size, the analysis was primarily descriptive. NATU Quest was administered only after completion of the intervention; therefore, no pre- to post-intervention inferential comparison of NATU Quest scores was performed.

No formal sample-size calculation was performed. This was an exploratory secondary analysis of all 15 participants who completed the parent study, and the sample was therefore determined by the parent cohort rather than by statistical power. The study was designed to describe usability rather than to test a hypothesis, and the findings should therefore be regarded as preliminary.

Open-ended responses were summarized descriptively according to the number of participants mentioning each recurring area of feedback. No formal qualitative analysis was performed.

## Results

Participant characteristics

Fifteen individuals with PD completed the two-week structured upper-limb rehabilitation programme and were included in the present usability analysis. The mean age of the participants was 67.07 ± 7.70 years. Ten participants were male (66.7%) and five were female (33.3%).

Most participants were classified as Hoehn and Yahr stage 2 (12/15, 80.0%), while two participants (13.3%) were classified as stage 3 and one participant (6.7%) as stage 1. Thirteen participants (86.7%) were right-hand dominant and two (13.3%) were left-hand dominant.

The baseline TETRAS ADL score was 21.53 ± 7.34, with higher scores indicating greater tremor-related difficulty in activities of daily living. All 15 participants completed the intervention and the post-intervention NATU Quest; no usability data were missing. The demographic and clinical characteristics of the participants are presented in Table 1 [9].

**Table 1:** Demographic and Clinical Characteristics of Participants (N = 15)

Variable	Value (Mean + SD/n(%))
Age (years), mean ± SD	67.07 ± 7.70
Baseline TETRAS ADL score, mean ± SD	21.53 ± 7.34
Sex, n (%)	
Male	10 (66.7%)
Female	5 (33.3%)
Hoehn and Yahr stage, n (%)	
Stage 1	1 (6.7%)
Stage 2	12 (80.0%)
Stage 3	2 (13.3%)
Hand dominance, n (%)	
Right	13 (86.7%)
Left	2 (13.3%)

All 15 participants completed the 10-item NATU Quest following completion of the two-week intervention. The mean overall NATU Quest score was 4.28 ± 0.32 out of 5.

Because NATU Quest has no validated cut-off for classifying usability as satisfactory, the scores are presented as observed participant ratings rather than interpreted against a predefined usability threshold. Among the individual NATU Quest items, ease of learning received the highest mean score (4.73 ± 0.46), followed by perceived safety and security (4.67 ± 0.49) and overall satisfaction (4.60 ± 0.51). Ease of use (4.53 ± 0.52) and willingness to recommend the device (4.47 ± 0.64) also received high scores.

The lowest mean score was observed for the physical comfort/fatigue item (3.33 ± 0.72). The remaining domains had mean scores ranging from 4.00 to 4.20.

The item-level NATU Quest findings are presented in Table 2. 

**Table 2:** The Item-Level NATU Quest Findings

NATU Quest item	Mean ± SD
Ease of use	4.53 ± 0.52
Meets individual needs	4.20 ± 0.56
Perceived safety and security	4.67 ± 0.49
Appropriate size, weight and dimensions	4.07 ± 0.88
Ease of interaction/control	4.20 ± 0.56
Physical comfort/no excessive fatigue	3.33 ± 0.72
Ease of learning	4.73 ± 0.46
Willingness to recommend	4.47 ± 0.64
Integration into daily lifestyle/environment	4.00 ± 0.76
Overall satisfaction	4.60 ± 0.51
Overall NATU Quest score	4.28 ± 0.32

Open-ended participant feedback

Open-ended responses indicated that participants generally perceived the glove as comfortable, safe, appropriately weighted, and easy to use. Six participants suggested reducing the glove weight or incorporating adjustable loading, particularly to facilitate prolonged use. Four participants recommended improvements in comfort or reduction of fatigue, while four participants suggested making the fastening mechanism easier to use, including easier application and removal. Three participants specifically suggested softer or improved padding, and three recommended better individual fit, adjustable sizing, or personalized fitting options. Regarding future applications, five participants expressed a preference for a glove that could be used during everyday or functional activities, while one participant specifically indicated a desire for independent use. Safety was identified as an important characteristic by five participants. These responses were considered descriptive feedback rather than a formal qualitative thematic analysis (Table 3).

**Table 3:** Descriptive Summary of Open-Ended Participant Feedback on the Prototype Weighted Glove

	Important design/manufacturing characteristics	Suggested changes to the existing glove	Desired future products/features
P1	Comfortable material and appropriate weight	Reduce the weight of the glove	Lighter glove for prolonged use
P2	Good fit, safety, and ease of use	Improve the fit around the hand	Adjustable-size glove
P3	Comfortable padding and manageable weight	Add softer padding	More comfortable glove for daily use
P4	Appropriate fit and easy fastening	Make fastening easier	Easier-to-wear rehabilitation glove
P5	Safety, comfort, and ease of handling	Improve comfort during prolonged use	Glove suitable for everyday activities
P6	Appropriate weight and comfortable design	Reduce fatigue during use	Adjustable-weight glove
P7	Secure fitting and ease of use	Improve fastening mechanism	Easier application and removal
P8	Appropriate size, fit, and weight	Improve individual fit	Glove available in different sizes
P9	Comfort, safety, and appropriate dimensions	Improve padding	Lighter and more comfortable glove
P10	Appropriate weight and ease of use	Allow adjustment of glove weight	Adjustable loading according to individual needs
P11	Comfortable material and good fit	Improve comfort and reduce fatigue	Lightweight glove for independent use
P12	Safety, comfort, and ease of use	Reduce physical fatigue	Glove usable during functional activities
P13	Individual fit and appropriate weight	Improve fit and fastening	Personalized glove according to individual requirements
P14	Comfortable design and manageable weight	Reduce weight and improve padding	Lighter glove for everyday use
P15	Appropriate fit, safety, and easy fastening	Make the glove easier to wear	More comfortable glove for daily functional activities

Safety results

All 15 participants completed the intervention, and no dropouts or adverse effects were reported during the two-week rehabilitation programme.

The lower physical comfort/no excessive fatigue rating (3.33 ± 0.72) was obtained from NATU Quest and should be interpreted as a participant-reported perception rather than as evidence of an adverse event.

## Discussion

Principal findings

The present study explored participant-reported usability, comfort, perceived safety, and satisfaction associated with a prototype weighted glove following two weeks of supervised upper-limb rehabilitation in people with PD. The overall NATU Quest score was 4.28 ± 0.32 out of 5, with high observed ratings for ease of learning, perceived safety and security, overall satisfaction, ease of use, and willingness to recommend the device. Because NATU Quest has no validated cut-off for satisfactory usability, these findings should be interpreted as observed participant perceptions rather than evidence that the device met a predefined usability threshold.

The lowest-rated item was physical comfort/fatigue (3.33 ± 0.72), identifying it as the comparatively least positively rated aspect of the prototype.

Ease of use and learning

Although these studies evaluated digital rehabilitation rather than a weighted glove, their findings provide contextual support for the importance of straightforward interaction and learnability in rehabilitation technologies. These comparisons should not be interpreted as evidence that the prototype weighted glove has equivalent usability to these technologies.

Perceived safety and satisfaction

Perceived safety and security (4.67 ± 0.49) and overall satisfaction (4.60 ± 0.51) were among the highest-rated domains.

These findings indicate high perceived safety and satisfaction during the supervised two-week programme. However, they should not be interpreted as evidence of long-term device safety or safety during independent use. The study had a small sample, was conducted under therapist supervision, and included no comparator or independent follow-up.

A recent pilot study of the CUE1 wearable device also reported positive comfort and usability findings [13]. Such comparisons provide contextual information regarding the importance of usability in wearable rehabilitation technologies but do not establish equivalence between devices.

Comfort and fatigue

Physical comfort and no excessive fatigue received the lowest observed NATU Quest rating (3.33 ± 0.72). This finding identifies comfort and fatigue as comparatively less positively rated aspects of the prototype but does not establish the presence of clinically significant fatigue or an adverse event.

Recent research involving wearable technologies in PD has similarly identified practical characteristics such as fit, adjustability, robustness, and physical prominence as relevant to user experience [14].

The open-ended responses in the present study provide additional context. Participants requested lighter or adjustable loading, improved fit, softer padding, easier fastening, and greater comfort. These observations identify practical design considerations for future development.

User-centred design considerations

The open-ended responses suggest several areas that may warrant further prototype development. Six participants requested a lighter or adjustable-weight glove, four requested greater comfort or less fatigue, four requested easier fastening, three requested softer or improved padding, and three requested improved or adjustable fit.

A 2026 study evaluating an assistive cutlery prototype for people with PD similarly demonstrated the value of user-centred design in addressing specific functional difficulties and evaluating usability and satisfaction [15].

The suggestions identified in the present study should, however, be regarded as design hypotheses rather than evidence that these modifications will necessarily improve usability. Future prototype studies should experimentally evaluate whether adjustable loading, improved fit, padding, or fastening mechanisms improve comfort and independent use.

Integration into daily life

The relatively lower ratings for appropriate size, weight, and dimensions (4.07 ± 0.88) and integration into daily lifestyle and environment (4.00 ± 0.76) may also be relevant to future development.

A systematic review of adoption of mobility assistive products identified user-centred design, challenges encountered during use, environmental factors, and training as factors influencing acceptance and continued use [16].

Although this literature is not specific to weighted gloves or PD, it supports the importance of considering how assistive devices fit into real-world environments rather than evaluating them only during supervised clinical use.

Distinction between usability and clinical effectiveness

The present findings concern participants’ experience of the prototype and do not address its therapeutic effectiveness, which was examined separately in the parent study [8]. These two types of evidence answer different questions. A device may be clinically promising but difficult or uncomfortable to use, whereas a highly usable device may not necessarily produce meaningful clinical benefits. Therefore, usability findings should complement rather than replace clinical effectiveness outcomes.

Glove weight and individualization

An important consideration is the external loading used in the present study. Participants used 600, 650, or 700 g according to the supervising physiotherapist’s assessment of comfort, physical capacity, quality of movement, and participant feedback. The selected weight was maintained throughout the two-week programme.

Participants nevertheless requested lighter or adjustable loading in the open-ended responses. This suggests that individual differences in strength, tremor severity, endurance, body size, and tolerance to external loading may be relevant to future device development. However, the present study was not designed to compare different loading strategies and therefore cannot determine whether lighter or adjustable loading is superior.

Relationship with the parent study

The present analysis adds a participant-experience perspective to the previously published clinical investigation from the same cohort [8]. The combination of relatively high overall satisfaction with a lower comfort rating suggests that participants generally reported positive experiences while identifying specific physical characteristics that could be refined.

These findings should therefore be viewed as complementary usability information rather than as evidence of therapeutic efficacy.

Strengths and limitations

Strengths

A strength of this study is the assessment of multiple dimensions of participant experience using a questionnaire specifically developed for people with neurological conditions [7]. The NATU Quest data were collected prospectively as a prespecified secondary outcome in the parent study. In addition, open-ended participant feedback allowed participants to identify specific device characteristics that could be considered during subsequent prototype development.

Limitations

Several limitations should be considered. First, the sample was small (N = 15), and no formal sample-size calculation was performed because the present analysis included the complete parent-study cohort rather than being powered for hypothesis testing.

Second, the analysis included only participants who completed the parent study. Although all 15 enrolled participants completed the intervention and NATU Quest, the perceptions of individuals who might have discontinued the intervention were unavailable; therefore, selection bias cannot be excluded.

Third, NATU Quest was administered only once, after the two-week intervention. Changes in usability perceptions over time could therefore not be evaluated.

Fourth, there was no comparator or control device. Consequently, the present study cannot establish that the prototype was more usable or acceptable than another rehabilitation device or intervention.

Fifth, 12 participants completed NATU Quest independently and three required the items to be read aloud because of motor limitations. Although responses were recorded without prompting, differences in administration mode may have influenced questionnaire responses.

Sixth, no specific procedures were used to minimize social-desirability bias. Ratings were obtained at the end of a therapist-supervised rehabilitation programme, which may have encouraged more positive responses.

Seventh, the prototype was evaluated only during short-term supervised use. Its usability, safety, and acceptability during prolonged independent use or routine daily activities remain uncertain.

Eighth, the present study was conducted as part of a multimodal rehabilitation programme. Therefore, the participant ratings cannot establish whether perceptions were attributable specifically to the weighted glove or to the overall rehabilitation experience.

Ninth, the study was not designed to determine the optimal external load. Although loading was selected according to participant-specific comfort, capacity, movement quality, and verbal feedback, no standardized loading-titration procedure was evaluated.

Tenth, disease duration, medication dose and timing, comorbidities, body weight and anthropometric measures, and upper-limb function other than the TETRAS ADL subscale were not recorded in the parent study.

Finally, pain, posture, gait, and other musculoskeletal factors were not assessed and may have influenced participants’ comfort or tolerance of the glove. Participants with severe joint pathology or an acute upper-limb injury were excluded, so the findings may not apply to people with such problems.

Future directions

Future studies should evaluate the prototype in larger samples and over longer periods, including periods of independent use. Future prototype development should test adjustable loading, fit, padding, and fastening in larger studies to determine whether these modifications improve comfort and independent use.

Comparative studies could evaluate different loading strategies and glove configurations, while combining participant-reported usability with objective functional outcomes. Such studies may help determine whether favourable user experience is associated with sustained use and clinically meaningful functional outcomes.

## Conclusions

In this small exploratory cohort, the prototype weighted glove received generally high user-reported usability and satisfaction ratings after two weeks of supervised use. The highest observed ratings concerned ease of learning, perceived safety and security, ease of use, and overall satisfaction, whereas physical comfort and no excessive fatigue received the lowest ratings.

These findings describe short-term participant perceptions and should not be interpreted as evidence of clinical efficacy, superiority over other interventions, or safety during independent use. Future prototype development should test adjustable loading, fit, padding, and fastening in larger studies to determine whether these modifications improve comfort and independent use.

## Figures and Tables

**Figure 2:**
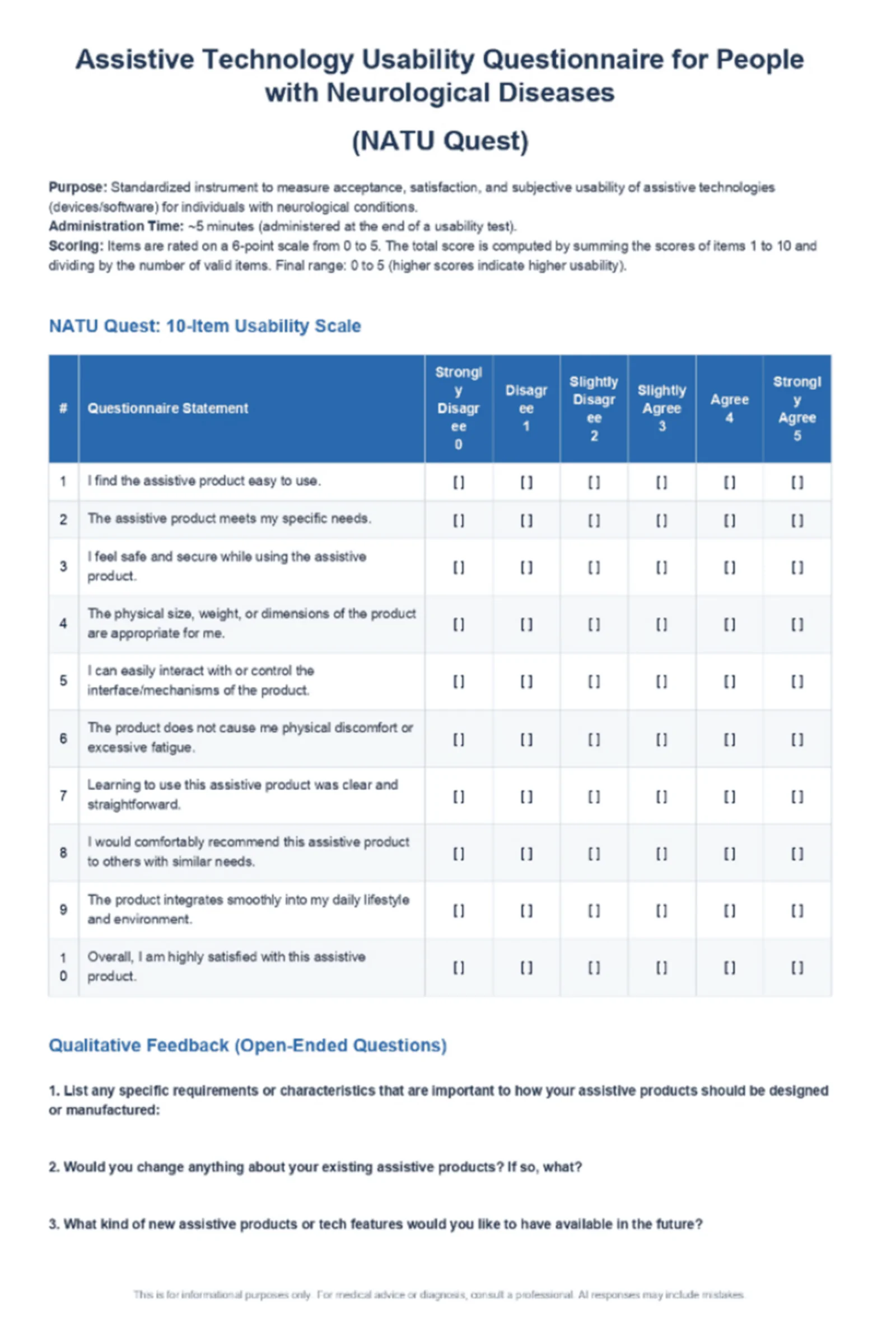
NATU Quest Source: Masbernat-Almenara et al. [8] under the Creative Commons Attribution 4.0 International (CC BY 4.0) licence

## Appendices

Appendix 
